# Activity May Not Reflect the Numbers: An Assessment of Capture Rate and Population Density of Dingoes ( *Canis familiaris* ) Within Landscape‐Scale Cell‐Fencing

**DOI:** 10.1002/ece3.71328

**Published:** 2025-04-27

**Authors:** Moses I. Omogbeme, Malcolm S. Kennedy, Tracey L. Kreplins, Halina T. Kobryn, Patricia A. Fleming

**Affiliations:** ^1^ Centre for Terrestrial Ecosystem Science and Sustainability, Harry Butler Institute Murdoch University Murdoch Western Australia Australia; ^2^ School of Environmental and Conservation Sciences Murdoch University Murdoch Western Australia Australia; ^3^ Department of Animal and Environmental Biology, Faculty of Life Sciences University of Benin Benin City Nigeria; ^4^ Department of Primary Industries and Regional Development Perth Western Australia Australia; ^5^ Department of Environment, Tourism, Science and Innovation Brisbane Queensland Australia; ^6^ Department of Primary Industries and Regional Development Northam Western Australia Australia

**Keywords:** exclusion fence, livestock, population density, predator control, spatially explicit capture–recapture, wild dog

## Abstract

Most human‐carnivore conflicts arise from the impact of predation on livestock. In Australian rangelands, considerable resources are allocated to constructing exclusion fences and implementing control measures to manage dingo populations for sustainable livestock enterprise. Assessing the effectiveness of these measures is crucial for justifying the investment. We used a replicated experimental design to examine the effect of landscape‐scale dingo‐proof exclusion fences (‘cell‐fencing’) on activity and population density of dingoes in the Southern Rangelands of Western Australia. We monitored dingo populations for 22–24 months across six study sites nested within a landscape of about 75,000 km^2^ and defined ‘fence level’ as the number of dingo‐proof fences enclosing each study site. We used camera trap capture rate (number of independent capture events per 100 trap nights) as a metric for dingo activity (including the availability of resources as other potential covariates), estimated dingo density using spatially explicit mark‐resight models, and tested the relationship between capture rate and estimated density of dingoes for each study site. Significant variation in both metrics was observed between sites and across time. Fence level and prey occurrence significantly influenced dingo activity. The annual mean dingo density estimate across study sites was below two dingoes per 100 km^2^ (i.e., 0.02 dingoes per km^2^; the maximum value believed to be compatible with small livestock) at only one study site in the first year, but it was higher across all sites during the second year of monitoring. Dingo activity correlated with estimated dingo density at only two sites, suggesting differences in dingo behaviour and detection across the six study sites. This study provides experimental evidence that camera trap capture rate is not a reliable method for assessing variations in the population size of dingoes. These results have implications for monitoring outcomes of dingo control programs across Australia.

## Graphical abstract

This study addresses a critical question: How reliable is the activity metric (here we used camera trap capture rate) to assess variations in population size of dingoes across diverse habitats in Australia? Through experimental evidence, we demonstrate that activity metrics can lead to inaccuracies in assessing changes in dingo population size. Our findings highlight the need to assess dingo population size from identified individuals, which is particularly relevant for effective dingo control programs across Australia.

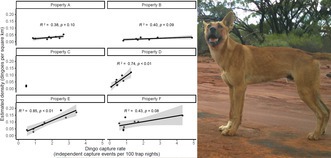

## 1 Introduction

Globally, their impact on livestock and subsequent conflict with humans places large carnivores at significant risk of extirpation (van Eeden et al. 2018). This has been the case for grey wolves ( *Canis lupus* ) and coyotes ( *Canis latrans* ) in North America and Europe (Treves and Karanth 2003; Knowlton et al. 1999; Fritts et al. 2003), black‐backed jackals ( *Canis mesomelas* ) and caracals ( *Caracal caracal* ) in South Africa (Kerley et al. 2018; Minnie et al. 2016), and dingoes ( *Canis familiaris* ) in Australia (Fleming et al. 2006; Allen 2016). Fencing is one of the most widely used methods to reduce predation of livestock (Moreira‐Arce et al. 2018), protecting small areas where animals are kept at night (Bauer et al. 2010) through to large‐scale protection of grazing areas (Smith et al. 2020). Fencing can be an effective and efficient way to reduce livestock losses to predators (Moreira‐Arce et al. 2018; van Eeden et al. 2018; Khorozyan and Waltert 2019; Miller et al. 2016), although its success depends on the agricultural system (Eklund et al. 2017).

Dingoes occupy at least 85% of mainland Australia (Newsome et al. 2015). In grazing areas, dingoes directly kill livestock but also cause injuries and indirectly affect livestock through exclusion from resources such as waterpoints (Fleming et al. 2014). Small livestock (e.g., sheep) are at the greatest risk from dingo predation impacts. It has been argued that sheep cannot coexist with dingoes due to predation (Fleming et al. 2001; Fleming 1996b), and dingo predation has forced the withdrawal from sheep production and a shift in livestock enterprise choice across much of the Australian rangelands (Allen and West 2013; Forsyth et al. 2014; Allen and West 2015). Thus, highlighting the need for exploration of a range of alternative management options.

Enormous effort and capital in excess of AUD$230 million per annum are expended in the control of dingo populations to maintain viable livestock enterprises across Australian rangelands (Hafi et al. 2023). Over the last century, landscape‐scale fences have been used to exclude dingoes from valuable agricultural areas (Glen and Short 2000; McKnight 1969; Prowse et al. 2015; Fleming et al. 2006), with shooting, trapping, and broadscale spread of poisoned baits to control dingo numbers inside the fenced areas. New dingo exclusion fences are currently being built, and old fences are fortified, but little is known about their effectiveness in assisting the management of the dingo population. The efficiency of any predator‐exclusion fence is a function of its size, design, permeability, effective maintenance, where it is located, and efficacy of control inside the fenced area (Bode and Wintle 2010; Norbury et al. 2014; Pacioni et al. 2021). Quantifying the effect of dingo exclusion fences and control action on dingo populations is therefore important in informing whether the investment in these fences and control is effective.

**FIGURE 1:**
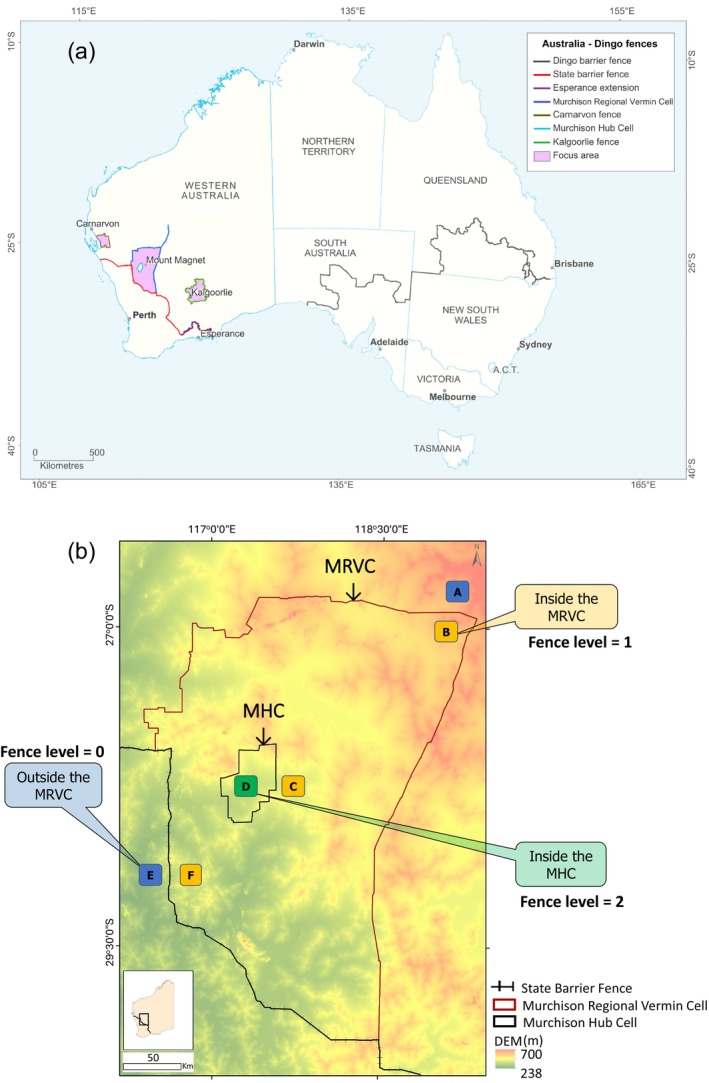
(a) Map of Australia showing the areas of recent investment in dingo exclusion fences (“Focus area”) in the Southern Rangelands of Western Australia (WA), including the Murchison Regional Vermin Cell (MRVC) and the Murchison Hub Cell (MHC) (Department of Primary Industries and Regional Development 2019). (b) Map of the study area with respect to elevation (generated from a 30 m resolution digital elevation model) showing six study sites (properties A, B, C, D, E, and F) nested within three dingo exclusion fence levels (outside the MRVC, inside the MRVC and inside the MHC) in the Murchison Region of the Southern Rangelands, Western Australia (WA).

To mitigate the economic losses due to the impact of dingoes on livestock, in 2018, the Western Australia (WA) state government allocated AUD 4 million in funding, matching over AUD 4 million in‐kind contributions, for the construction and fortification of four dingo exclusion fences across the Southern Rangelands of the state (Carnarvon, Kalgoorlie, two in the Murchison Region—“Focus area”; Figure 1a). The present study was carried out within the two fenced cells in the Murchison Region in the Southern Rangelands of WA (Figure 1b): the Murchison Regional Vermin Cell (hereafter ‘MRVC’) and the smaller fenced area in the middle of the MRVC (the Murchison Hub Cell, hereafter ‘MHC’) to understand dingo responses to their management (exclusion fencing and population control). In addition to anthropogenic influence through exclusion fencing and population control, dingo populations are also likely to be affected by the availability of resources such as prey availability and access to drinking water. This study investigated the effect of landscape‐scale dingo exclusion on dingo activity and population density in the Southern Rangelands of WA, using 24 months of camera trapping surveys along vehicular dirt tracks to address the following research questions:

- Is dingo activity (camera trap capture rate) correlated with resource availability (prey species and access to drinking water) and exclusion fencing?

Dingoes are generalist predators, but they primarily hunt large mammals, predominantly macropods (Fleming et al. 2022), and also livestock, particularly in grazing areas (Duncan et al. 2022). Although dingoes presumably meet their water requirements from the blood and body fluid of prey, they frequently drink from water sources during hot and dry weather conditions (Allen 2012a; Corbett 1995b). During these periods, the distribution of water sources is strongly related to where prey species and dingoes are found, as dingoes focus their hunting around water sources to increase their frequency of encounter with prey species (Thomson 1992c; Brawata and Neeman 2011).

ii Do dingo activity (camera trap capture rate) and derived density estimates vary between study sites and over time?

The six study sites selected for this study represent different land use and management. The sites also represent three levels of dingo exclusion fencing, from no exclusion fencing to two exclusion fences (Figure 1b).

Dingoes show seasonal breeding and variation in activity, with greatest movements around breeding season (mid‐March to early June) (Thomson 1992b). The length of time that this survey was carried out over (24 months) allowed investigation of variation in dingo activity and estimated density over time, which would not be possible for a study of shorter duration.

iii Is dingo management in the Southern Rangelands of WA effective for maintenance of small livestock?

Pacioni et al. (2021) predicted that predator‐exclusion fences can have a significant effect on dingo density, assuming minimal permeability (< 1%) and if the initial dingo density is less than two dingoes per 100 km^2^, or where there is low survival of control (≤ 0.25). We therefore set out to determine whether management was successfully suppressing annual variation in dingo density and whether dingo populations were maintained below this desired density.

iv Are dingo activity (camera capture rate) and population density estimates correlated?

Accurate assessment of dingo populations and the effects of management on dingo populations can be challenging. Dingoes are cryptic and occupy large home ranges ranging from < 1 km^2^ (McNeill et al. 2016) to 2000 km^2^ (Newsome et al. 2013), making a robust assessment of their activity and population size challenging. As such, the assessment of management effects on dingo population has been largely limited to estimates derived from the count of dingo signs (e.g., footprint and faeces) (Corbett 1995a; Fleming et al. 1996; Newsome et al. 1972). The application of heat‐in‐motion camera traps that provide sufficient photo quality to identify individuals, combined with spatially explicit mark‐resight (SEMR) statistical models [an extension of spatially explicit capture–recapture (SECR) methods], allows estimation of population density from resighting known individuals. This method has been used to investigate dingo population densities along a peninsula in northern Queensland (Gabriele‐Rivet et al. 2020), or the effect of poisoned baits as a control tool on dingo population density in pastoral properties in WA (Kennedy et al. 2021). However, there has been no published comparison of dingo activity and density at landscape‐scale cell‐fencing.

## 2 Materials and Methods

This research was conducted under the Murdoch University ethics approval (Protocol ID. 711, Permit No. O3214/20). Permission for field work on study sites was granted by the WA state Department of Biodiversity, Conservation and Attractions (DBCA) and pastoral lease owners. Permission to drive along the State Barrier Fence (SBF) which separates the rangelands from southwest WA was granted by the WA state Department of Primary Industries and Regional Development (DPIRD).

### 2.1 Study Site Description

The MRVC (75,000 km^2^) encloses 52 pastoral stations. Central within the MRVC, the MHC encloses four pastoral stations. The study sites were categorized by their fence level: outside MRVC (fence level 0), inside the MRVC (fence level 1), and inside the MHC within the MRVC (fence level 2) (Figure 1b). The six properties captured different forms of land management. Although most pastoral stations within the MRVC have a history of sheep farming, there has been a shift in enterprise composition over time in terms of livestock choice and numbers. At the time of this study, four of the study sites (properties A, B, C, and D) were pastoral stations and the other two study sites (properties E and F) were within the west and east portions of a Rangelands Park (ex‐pastoral station, but de‐stocked for conservation purposes).

At the time of this study, dingoes were controlled across all study sites. In addition to dingo exclusion fencing, broadscale bi‐annual coordinated ground‐deployment of poisoned 1080 (sodium fluoroacetate) baits is generally undertaken across all of these properties by the Regional Biosecurity Groups (RBGs), who also employ trapping and opportunistic shooting to control dingo populations (Table 1). This control has been carried out as per legislative requirements to manage dingoes as a declared pest species for pastoral lands under the Biosecurity and Agriculture Management Act 2007 (BAM Act; https://www.agric.wa.gov.au/).

**TABLE 1:** Control effort employed to reduce dingo population and impact across the six study sites (properties A, B, C, D, E, and F) nested within three dingo exclusion fence levels (outside MRC, inside MRVC, and inside MHC) in the Murchison Region of the Southern Rangelands, Western Australia (WA).

Location within the MRVC	Study site (property)	Managed livestock (unmanaged)	Exclusion fencing		Lethal control technique					Waterpoints (number of man‐made bores)
			Predator‐proof fence	Fence level (number of fences enclosing the study site)*	Bi‐annual baiting	Targeted baiting	Trapping	Opportunistic shooting	Lethal control score*	
Northeast	A	Cattle	N (outside MRVC)	0	Y	Y	Y	Y	4	Present (40)
	B	Cattle	Y (inside MRVC)	1	Y	Y	Y	Y	4	Present (42)
Central	C	Cattle (Goats)	Y (inside MRVC)	1	Y	Y	Y	Y	4	Present (< 10)
	D	(Goats)	YY (inside MHC & MRVC)	2	Y	Y	Y	Y	4	Present (< 10)
Southwest	E	(Cattle)	N (outside MRVC)	0	Y		Y		2	Decommissioned
	F	(Cattle)	Y (inside MRVC)	1	Y		Y		2	Decommissioned

Abbreviations: MHC = Murchison Hub Cell, lying centrally in the MRVC; MRVC = Murchison Regional Vermin Cell; N = Nil; Y = Yes.

* Summed scores for exclusion fences and lethal control techniques.

Properties A and B were in the northeast corner of the MRVC, with property A (1,565 km^2^) outside the MRVC (fence level 0) and property B (1,494 km^2^) inside the MRVC (fence level 1). At the time of the study, both properties were entirely commercial cattle grazing and were equipped with A:40 and B:42 functioning artificial bores providing water for livestock.

Properties C and D were located centrally in the MRVC, with property C (1065 km^2^) outside the MHC (but still inside the MRVC, fence level 1) and property D (1494 km^2^) inside the MHC (fence level 2). A herd of cattle and unmanaged goats grazed the west end of property C while unmanaged goats grazed the north of property D. At the time of this study, properties C and D each had less than 10 functioning man‐made bores providing water to the surface.

The Rangelands Park (5607 km^2^) lies in the southwest of the MRVC. The SBF runs north–south through the middle of the Rangelands Park, forming the west portion (property E; outside the MRVC, fence level 0) and east portion (property F; inside the MRVC, fence level 1). The Rangelands Park had been de‐stocked for over 20 years, although some feral livestock (cows) persisted. All the bores in the Rangelands Park were decommissioned; however, there were a few lakes and creeks from which wildlife could drink for most of the year.

Within each study site, camera traps were deployed across the entire property area. Sites A and B were on either side of the fence separating them, as were sites E and F, while the two central study sites were separated by a 70 km distance. There was about 400 km between the northeast and the southwest sites (Figure 1b).

The Southern Rangelands of WA are characterised by arid/semi‐arid environments. From the northeast to the southwest of the MRVC, mean annual rainfall increases from 215 mm to 345 mm, with a slight decrease in mean maximum temperature from 38.4°C to 37.4°C in January (Bureau of Meteorology 2023). The vegetation across the study area is composed of dense tall shrubs and perennial grasses (Burnside et al. 1995) of mostly *Acacia* spp., which become sparsely distributed from the southwest to the northeast of the MRVC.

### 2.2 Experimental Design

Before‐After Control‐Impact (BACI) experimental design could not be implemented in this present study because pastoral lease owners are required under legislation (BAM Act) to control dingo populations across all land tenures in the study area. Moreover, dingo management across the Southern Rangelands of WA has been ongoing for over 100 years. As such, our experimental design was correlative (using stratified randomised survey points) and could not include experimental control sites. Thus, sampling surveys were done across study sites as pseudo‐experiment type 1, with an “H Score” (experimental design strength) of 9 (Hone 2007; Castle et al. 2023).

We used ArcGIS software (version 10.6) to analyse terrain rugosity and vegetation data to identify suitable road access and sampling transects at each study site. The study was designed to avoid significant areas of breakaways, spinifex, and salt lakes, to minimise inter‐site environmental variation in geology, understory vegetation type, and standing surface water (details in Appendix S1 and Table S1). We monitored six replicate 5 km‐long transects along low‐use vehicular dirt roads in each study site (a total of 36 sampling transects across the study area). Our sampling transects were a minimum of 1.5 km from waterpoints (lakes, creeks, artificial boreholes, or water tanks), and avoided the dingo‐proof fences to reduce bias due to funnelling of animals. As a result, we spaced the transects at an average of 10 km apart (5–20 km) to cover a significant portion of each study site.

### 2.3 Camera Trap Monitoring

Each of the 36 transects included two camera traps, deployed 2 km apart from each other along vehicular dirt tracks. Camera traps were vertically orientated on a metal stake, 0.5 m above the ground and angled southwest to avoid false triggers by sun glare (after Meek et al. 2016) at approximately 22.5° to the direction of travel of the road. The 72 camera traps included three Reconyx models evenly distributed across each study site (HC500 Hyperfire, HC600 Hyperfire, and XR6 Ultrafire; Reconyx, Holmen, WI, USA). No lures or attractants were used for this study. The 72 camera traps operated for 22–24 months from July/August 2020 to May/June 2022, for a total of 43,628 trap nights (mean: 605 trap nights per camera) and were serviced (to replace SD cards and batteries) at approximately three‐to‐five‐month intervals according to logistical and study site access constraints. We set the motion‐sensing cameras to high sensitivity and to take three consecutive photographs when triggered, with no quiet period between trigger events.

#### 2.3.1 Calculating Dingo Activity (Camera Capture Rate)

Camera trap images were viewed and classified using the Aegir model on eVorta Autonomous Vision (https://evorta.com/), an online artificial intelligence software for image processing and identification. All images of dingoes were downloaded from the eVorta platform as standalone folders for further individual identification purposes and viewed on digiKam (v. 7.9.0; DigiKam 2022). At each camera trap location, dingo images at greater than 10‐min intervals were considered independent capture events, following Kennedy et al. (2021). Dingo capture rate (independent capture events of dingoes per 100 trap nights) was calculated for each camera trap location per sampling month. Similarly, the capture rate for herbivores that used vehicular tracks (macropod, rabbit, livestock, goat, camel and horse) was also calculated from camera trap images.

#### 2.3.2 Calculating Dingo Density From Individual Identification

Spatial Capture‐Mark‐Resight estimation (Efford and Hunter 2018) was performed to estimate the density of dingoes present across the six study sites. Unique identification of dingoes is required for this approach. Camera trap images of dingoes were tagged using digiKam (v. 7.9.0; DigiKam 2022) and individual identification was carried out by three independent observers. We classified the images of dingoes as ‘identifiable marked’ if consensus was reached on their identity, ‘unidentifiable marked’ where consensus was not reached on their identity, or ‘unmarked’ where poor image quality precluded their identification. The ‘identifiable marked’ data was used to create a daily capture history of dingoes in each study site.

The density of dingoes was estimated for each study site by fitting multi‐session spatially explicit capture–recapture (secr) models to the daily capture history of dingoes, using the ‘secr’ package (v. 4.6.0; Efford and Jund 2023) in R (v. 4.2.3; R Core Team 2023). Daily capture history of identified individuals was binned into eight trap sessions (maximum 92 occasions; 1 occasion = 1 day) for each study site, to comply with the model assumption that the dingo populations were closed at 3‐month intervals (Table 2), following Gabriele‐Rivet et al. (2020). The exemption to this data handling was property C, where it was only possible to analyse data aggregated into yearly sessions (merging capture histories of sessions 1 to 4 as year 1, and sessions 5 to 8 as year 2) due to very low capture–recaptures. A habitat mask for each study site was fit into the secr models using the coordinates of each property boundary (Efford 2023), as this generally reflects fence lines.

**TABLE 2:** Trap sessions and occasions used for analysis of dingo density (spatially explicit mark‐resight analysis) across six study sites in the Murchison Region of the Southern Rangelands, Western Australia (WA).

Session	Months	Occasions (days)
1	July–September 2020	92
2	October–December 2020	92
3	January–March 2021	90
4	April–June 2021	91
5	July–September 2021	92
6	October–December 2021	92
7	January–March 2022	90
8	April–June 2022	91

In secr models, detectability is a function of two parameters:

- *g*0, the probability of detection (per occasion) when the distance between an animal's activity centre and a detector (camera trap) is zero, and
- *σ*, a spatial scale index reflective of home range.

Hence, the probability of detection declines when dingoes move away from their home range centre. This decline in detection probability was tested using four detection functions: half‐normal, hazard‐rate, exponential, and hazard half‐normal, with the best detection function selected as the model fit with the lowest AICc. The best‐selected model fit was then used to further test for a possible effect of learned response in dingoes to camera traps on *g*0; *g*0 ~ *b* (global learned response, i.e., a response to all camera traps) and *g*0 ~ *bk* (detector‐specific learned response, i.e., a response to each camera trap).

### 2.4 Statistical Analyses

#### 2.4.1 Is Dingo Activity (Camera Capture Rate) Correlated With Resource Availability (Prey Species and Access to Drinking Water) and Exclusion Fencing?

To examine the relationship between dingo activity and landscape management (exclusion fencing, water presence, and prey activity), a generalised linear mixed model (GLMM) was fit to the monthly dingo capture rate (rounded off to integers) as the response variable, with dingo exclusion fence level (0–2), water presence and potential prey activity (macropod, rabbit, livestock and feral ungulate camera trap rates) as predictor variables (Table 3), and camera trap location (‘cameraID’) as a random factor to account for repeated measures over time. Data exploration for linear regression (response and predictor variables) was carried out following Zuur et al. (2010). The highest variance inflation factor (vif) for the six predictor variables was less than 1.12 (vif for water presence), which indicated no issues with collinearity between the predictor variables. All continuous predictor variables were mean standardised (centred at their means and divided by their standard deviations) in R. Due to the high number of true zeros (76%) and statistical dispersion in the response variable, Quasi‐Poisson Regression was selected as the best model fit for the data.

**TABLE 3:** Variables across camera trap locations used to examine the relationship between dingo capture rate (number of independent capture events per 100 trap nights) and landscape management across six study sites in the Murchison Region of the Southern Rangelands, Western Australia (WA).

Variable	Description and source
Response variable	
Dingo CR	Capture rate (CR) for dingoes was calculated as the number of independent capture events (10 min independent threshold) of dingoes at each camera trap location divided by the total number of days in which camera traps were active for each survey month multiplied by 100. Rounded to integers for negative binomial linear regression analysis
Explanatory variable	
Dingo exclusion fence (Fence level: 0–2)	Dingo exclusion fence was categorised as outside MRVC (0), inside MRVC (1), and inside MHC (2), and used as a categorical variable for linear regression analysis
Water presence [Present (1)/Absent (0)]	Existing terrain rugosity data for on‐site analysis of the landscape to identify suitable sampling transects in each study site using Arc GIS software (version 10.6), was used to calculate the distance of camera traps to the closest permanent water source (lake, artificial boreholes, water tank). This was followed by ground truthing during camera trap deployment. The presence (1) or absence (0) of a water source within a 5 km radius of each camera trap location was used as a categorical variable for linear regression analysis
Macropod CR	Capture rate for macropods was calculated similarly to dingoes. Continuous variable, log‐transformed for linear regression analysis
Rabbit CR	Capture rate for European rabbits ( *Oryctolagus cuniculus* ) was calculated similarly to dingoes. Continuous variable, log‐transformed for linear regression analysis
Livestock CR	The number of independent camera trap capture events (10 min independent threshold) of cow ( *Bos taurus* ) per 100 trap nights was used to calculate the capture rate for livestock, similar to dingoes. Continuous variable, log‐transformed for linear regression analysis
Feral ungulate CR	Number of independent camera trap capture events (10 min independent threshold) of combined feral goat ( *Capra hircus* ), feral horse ( *Equus caballus* ), and camel ( *Camelus dromedarius* ) records per 100 trap nights was used to calculate the capture rate for introduced feral ungulate. Continuous variable, log‐transformed for linear regression analysis

Abbreviations: MHC = Murchison Hub Cell, lying centrally in the MRVC; MRVC = Murchison Regional Vermin Cell.

The Template Model Builder (TMB), which uses maximum likelihood estimation and allows for pseudo‐replications of random effect (i.e., ‘cameraID’), was used to fit the GLMM with link function ‘*nbinom1*’, using the *glmmTMB* function in the ‘glmmTMB’ package (v. 1.1.7; Brooks et al. 2023) in R. The global model validation was confirmed using residual diagnostics for linear regression with the ‘DHARMa’ package (v. 0.4.6; Hartig and Lohse 2022) in R. The global model was tested for overdispersion and zero inflation, and the DHARMa scaled residuals were plotted against each continuous predictor variable. A dredge was performed to fit all possible combinations of variables in the global model using the ‘MuMIn’ package (v. 1.47.5; Barton 2023) in R. The model with the lowest Akaike's Information Criterion adjusted for a small sample size (AICc) was selected as the best fit. The *ggpredict* function in the ‘ggeffect’ package (v. 1.3.1; Lüdecke et al. 2023) in R, was used to predict dingo capture rate across fence level (outside MRVC, inside MRVC, and inside MHC), with the other predictor variables controlled at mean values.

#### 2.4.2 Do Dingo Activity (Camera Trap Capture Rate) and Derived Density Estimates Vary Between Study Sites and Vary Over Time?

A nonparametric Kruskal‐Wallis test in R was used to compare dingo activity between study sites and over time, using the monthly dingo capture rate (independent capture events per 100 trap nights) for each camera trap location as the dependent variable. Study site (‘Site’) and sampling month (‘Month’) were used as predictor factors in separate analyses.

To test for significant differences in the estimated density of dingoes between study sites, a linear mixed model (LMM) was fit to the secr density estimates across all sessions (dependent variable), with study site (‘Site’) as the predictor variable, and trap session (‘Session’; 1–8, ‘Year 1’, and ‘Year 2’ for property C) included as a random effect to account for repeated density estimates over time. To test for the effect of exclusion fencing and trap session on dingo density across the study sites, a second LMM was then fit to the secr density estimates (dependent variable), with dingo exclusion fence level (0–2) and trap session as the predictor variables, again accounting for repeated measures by including study site (‘Site’) as a random effect. The LMMs were fit using the *lmer* function in the ‘lme4’ package (v. 1.1‐33; Bates et al. 2023), and model validation was confirmed using the ‘DHARMa’ package in R. Where there was a significant effect (of study sites and/or fence level), the result was followed by pairwise post hoc (adjusted for Tukey) analysis (between study sites and/or fence level) using the ‘emmeans’ package (v. 1.8.6; Lenth et al. 2023) in R. From the output of the second LMM, dingo density estimate (dingoes per km^2^) across fence levels was predicted using the *ggpredict* function in the ‘ggeffect’ package in R.

#### 2.4.3 Is Dingo Management in the Southern Rangelands of WA Effective for Maintenance of Small Livestock?

To address this question, we identified which sessions the estimated density of dingoes was below the level of 2 dingoes per 100 km^2^ (Pacioni et al. 2021). Variation in the mean annual population density of dingoes was estimated for each study site. Dingo density was calculated as mean values of density estimates in the first year (12 months) of monitoring, from session 1 (July 2020) to session 4 (June 2021) at each study site. Mean values of dingo density estimates were also calculated for the second year of monitoring from session 5 (July 2021) to session 8 (June 2022).

#### 2.4.4 Are Dingo Activity (Camera Capture Rate) and Population Density Estimates Correlated?

To test the relationship between dingo activity (i.e., capture rate: number of independent capture events per 100 trap nights) and population density (dingoes per km^2^) in each study site, linear regression was performed. To do this, the dingo capture rate was calculated for each study site for the eight trap sessions (three‐month intervals; Table 2) from the ‘identifiable marked’ data used for dingo density estimates. With the exemption of property C (where dingo density estimates were only possible for yearly sessions), linear regression models were fitted in R using *lm* function to test the correlations between the dingo capture rate and estimated density for each study site. Model validations were confirmed using the ‘DHARMa’ package in R.

## 3 Results

Dingoes were detected on 93% of camera trap locations over the 24 months of monitoring. In all capture events of dingoes at each camera trap location, the first and last images of the same series were less than 5‐min intervals, indicating the 10‐min interval for consideration of trap captures as independent was conservative. Independent capture events ranged between 2 h to 9 months intervals on the same camera trap location. A total of 587 independent capture events yielded 800 dingo detections (marked and unmarked). About 98% (780 out of 800) of dingo detections across the six study sites were identifiable to individual, with 292 identified individuals recorded across all six study sites (Table 4). Almost half (128; 44%) of the 292 identified individuals were sighted at more than one camera trap location. The number of dingo detections, identifiable detections, identified individuals, and the percentage of identified individuals sighted at more than one camera trap location varied between study sites (Table 4). There was no evidence of identified individuals moving between sites.

**TABLE 4:** Summary of camera trap survey data for dingoes from six study sites (properties A, B, C, D, E, and F) nested within three dingo exclusion fence levels (outside MRC, inside MRVC, and inside MHC) in the Murchison Region of the Southern Rangelands, Western Australia (WA).

	Northeast		Central		Southwest		Total
Study site (property)	A	B	C	D	E	F	
Fence level	Outside MRVC	Inside MRVC	Inside MRVC	Inside MHC	Outside MRVC	Inside MRVC	
Number of camera trap locations	12	12	12	12	12	12	72
Camera trap locations where dingoes were detected (%)	100%	100%	75%	92%	92%	100%	93%
Number of identifiable marked dingo detections	191	173	45	49	210	112	780
Number of unidentifiable marked dingo detections	0	0	0	0	3	2	5
Number of unmarked dingo detections	6	4	1	0	3	1	15
Number of identified individuals	66	65	14	31	66	50	292
Number of identified individuals sighted at more than one camera trap location	41	29	2	4	35	17	128
Percentage of identified individuals sighted at more than one camera trap location	62%	45%	14%	13%	53%	34%	44%
Mean ± SD [95% CI] dingo density (number of dingoes per km^2^)*	0.031 ± 0.011^*a*^, [0.017–0.055]	0.022 ± 0.009^*b*^, [0.012–0.041]	0.019 ± 0.005^*ab*^, [0.008–0.047] †	0.057 ± 0.037^*ab*^, [0.020–0.170]	0.098 ± 0.060^*b*^, [0.057–0.170]	0.090 ± 0.039^*b*^, [0.039–0.211]	

*Note:* Letters *a*, *b*, and *ab* are the results of pairwise comparisons linking sites where the dingo density estimates were not significantly different. The hazard‐rate function was applied as the best model for five study sites, except for property D where the exponential function provided the best model for secr analysis. There was no effect of the learned response of dingoes to camera traps (either global or detector‐specific) on g0 for the top‐rank model for each study site.

Abbreviations: MHC = Murchison Hub Cell, lying centrally inside the MRVC; MRVC = Murchison Regional Vermin Cell.

* Due to low numbers of individuals and low numbers of repeat sight dingoes, density estimates for Property C were based on two periods (1 year each) rather than eight 3‐monthly periods as was done for the other sites.

### 3.1 Is Dingo Activity Correlated With Resource Availability and Exclusion Fencing?

GLMM analysis used to examine the relationship between dingo activity (independent capture events per 100 trap nights, calculated by month of study) and six predictor variables (i.e., the ‘global model’) showed a significant effect (*p* < 0.05) of four predictor predictors (Table 5). The best model fit (with the lowest AICc) from all possible combinations of the predictor variables in the global model also had the same four variables.

**TABLE 5:** glmmTMB analysis outcome of the global model for dingo activity in the Murchison region of the Southern Rangelands, Western Australia (WA) during 24 months of monitoring. The shaded area depicts predictors with significant *p*‐values, with the *p*‐value in bold. CR = capture rate (number of independent camera trap capture events per 100 trap nights); MHC = Murchison Hub Cell, lying centrally in the MRVC; MRVC = Murchison Regional Vermin Cell.

Predictor variable	Estimate	SE	*p*
Intercept	0.29	0.20	0.144
Fence level: inside MRVC	−0.29	0.21	0.165
Fence level: inside MRVC and MHC	−0.78	0.31	**0.013**
Macropod CR	0.15	0.06	**0.018**
Rabbit CR	0.13	0.07	**0.047**
Livestock CR	0.21	0.07	**0.004**
Feral ungulate CR	−0.02	0.06	0.784
Water presence	−0.10	0.22	0.665

**FIGURE 2:**
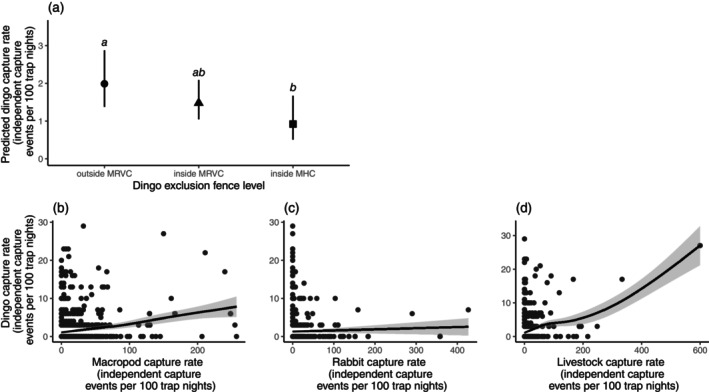
Four predictor variables that had significant effects on dingo capture rate in the Murchison region of the Southern Rangelands, Western Australia (WA) during 24 months of monitoring: (a) the relationship between predicted dingo capture rate and dingo exclusion fence level; the shapes indicate predicted mean values of dingo capture rate at fence levels, and the black bars depict 95% confidence intervals. The letters *a*, *b*, and *ab* indicate significant differences. MHC = Murchison Hub Cell, lying centrally in the MRVC; MRVC = Murchison Regional Vermin Cell. (b), (c) and (d) depicts the relationship between dingo capture rate and the capture rates of three prey groups.

Accounting for prey availability, dingo capture rate varied between fence levels (Figure 2). There was a 29% ± 21% decrease (*p* = 0.165) in dingo activity from outside the MRVC (mean 1.99 [95% CI 1.38–2.88] independent capture events per 100 trap nights) to inside the MRVC (1.48 [1.05–2.09] independent capture events per 100 trap nights), and a 78% ± 31% decrease (*p* = 0.013) from outside the MRVC to inside the MHC (0.92 [0.51–1.67] independent capture events per 100 trap nights).

### 3.2 Do Dingo Activity and Derived Density Estimates Vary Between Study Sites?

**FIGURE 3:**
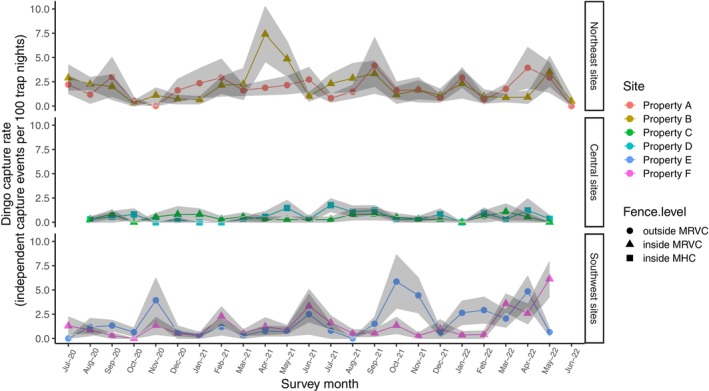
Dingo capture rate for 24 months in six study sites (properties A, B, C, D, E, and F) nested within three dingo exclusion fence levels (outside MRC, inside MRVC, and inside MHC) in the Murchison region of the Southern Rangelands, Western Australia (WA). Shapes depict monthly mean value summations from all camera trap locations in each study site and the fence level at which the site is categorized, while the colors of the shapes represent the study sites. Grey ribbons are standard error summations from all camera trap locations in each study site. MHC = Murchison Hub Cell, lying centrally in the MRVC; MRVC = Murchison Regional Vermin Cell.

Dingo activity varied significantly between study sites (*χ* ^2^_5_ = 60.47, *p* < 0.001; Figure 3). Overall, the highest dingo activity (mean ± SE capture events per 100 trap nights) was recorded in the northeast (property A: 1.83 ± 0.23, property B: 2.08 ± 0.26) and southwest sites (property E: 1.78 ± 0.26, property F: 1.28 ± 0.18), with least dingo activity for the central sites (property C: 0.47 ± 0.09, property D: 0.58 ± 0.11). However, there was no significant effect of sampling month on dingo activity (*χ* ^2^_23_ = 46.95, *p* > 0.05).

**FIGURE 4:**
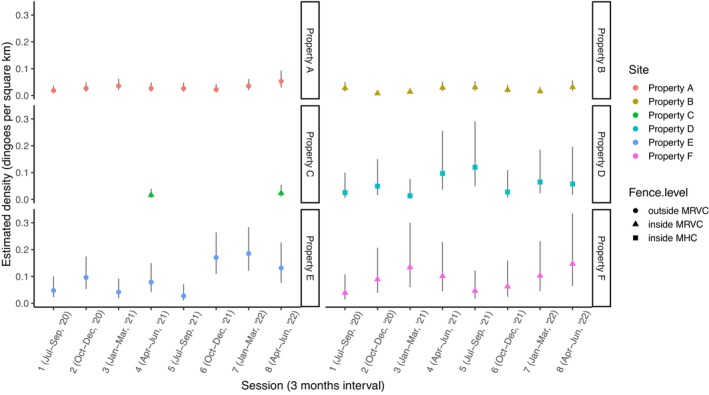
Estimated density of dingoes (3 months interval) in six study sites (properties A, B, C, D, E, and F) nested within three dingo exclusion fence level (outside MRC, inside MRVC, and inside MHC) in the Murchison region of the Southern Rangelands, Western Australia (WA) for 24 months of monitoring. Shapes depict mean values and the fence level at which the site is categorised, while the colours of the shapes represent the study sites. Black bars indicate 95% confidence intervals per trap session in each study site. MHC = Murchison Hub Cell, lying centrally in the MRVC; MRVC = Murchison Regional Vermin Cell.

The LMM analysis by study site indicated significant differences in dingo density between study sites (Figure 4). Pairwise post hoc analysis between sites indicated significant differences between the northeast sites (properties A and B) and southwest sites (properties E and F) (*p* < 0.05 for pairwise comparisons; Table 4). The central sites (properties C and D) with lower density estimates were also more variable, and therefore, pairwise comparison did not reach statistical significance.

**FIGURE 5:**
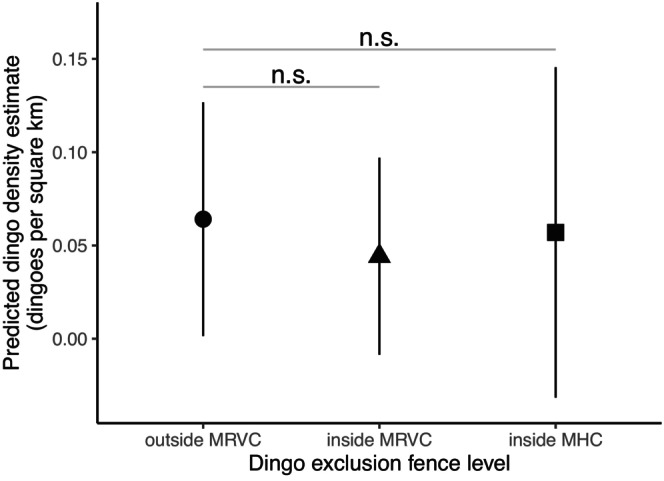
The relationship between predicted dingo density estimates and dingo exclusion fence level in the Murchison region of Southern Rangelands, Western Australia (WA) during 24 months of monitoring. The shapes indicate predicted mean values of dingo density estimate, and the black bars are 95% confidence intervals. The grey horizontal bars denote non‐significant (n.s.) *p*‐values. MHC = Murchison Hub Cell, lying centrally in the MRVC; MRVC = Murchison Regional Vermin Cell.

The LMM analysis by dingo exclusion fence and trap session indicated a significant influence of trap session (three‐month intervals, year intervals for Site C) on dingo density estimates (*t*_35_ = 2.65, *p* = 0.012), with a general increase in dingo density towards the later trap sessions of the study. There were also within‐annual patterns, with dingo density peaking about 3 months earlier for the southern sites compared with the northern sites (Figure 4). Estimated dingo density peaked during trap session 8 (between April to June 2022) at properties A and B. In the central sites, estimated dingo density was higher in the second year for property C (i.e., trap session 5 to 8, between July 2021 to June 2022), and peaked during trap session 5 (between July to September 2021) for property D. In the southwest sites, density estimates peaked during trap session 7 (between January to March 2022) at property E, and during trap session 8 (between April to June 2022) for property F. However, there was no significant effect of dingo exclusion fence level (from outside the MRVC to inside the MHC) on dingo density estimates (*t*_3.7_ = 0.28, *p* = 0.797). Predicted dingo density estimate (mean [95% confidence interval]) for outside the MRVC (mean 0.06 [0.00–0.13] dingoes per km^2^), inside the MRVC (mean 0.04 [−0.01–0.10] dingoes per km^2^), and inside the MHC (mean 0.06 [−0.03–0.15] dingoes per km^2^) is shown in Figure 5.

### 3.3 Is Dingo Management in the Southern Rangelands of WA Effective for the Maintenance of Small Livestock?

Annual mean dingo density across study sites was less than 2 dingoes per 100 km^2^ (i.e., 0.02 dingoes per km^2^) in the first year except for property C (1.6 dingoes per 100 km^2^ or 0.016 dingoes per km^2^; Table 6), but higher across all sites during the second year of monitoring. Changes in annual mean density estimates showed a general increase across study sites except for property F (i.e., the east portion of the Rangelands Park) where there was a 0.1% decrease. The highest annual increase in dingo density estimates was observed in property E (i.e., the west portion of the Rangelands Park; 6.3%) followed by property D (2.1%).

**TABLE 6:** Annual mean density estimate (dingoes per km^2^) and percentage change in dingo density for six study sites (properties A, B, C, D, E, and F) nested within three dingo exclusion fence levels in the Murchison region of the Southern Rangelands, Western Australia (WA).

Study site (property)	Survey	Months	Annual mean density estimate (dingoes per km^2^)	Δ Annual mean density estimate
A	Year 1 (initial)	12 (July ‘20 to June ‘21)	0.027	0.8%
	Year 2	12 (July ‘21 to June ‘22)	0.034	
B	Year 1 (initial)	12 (July ‘20 to June ‘21)	0.020	0.5%
	Year 2	12 (July ‘21 to June ‘22)	0.025	
C	Year 1 (initial)	11 (Aug ‘20 to June ‘21)	0.016	0.7%
	Year 2	11 (July ‘21 to May ‘22)	0.023	
D	Year 1 (initial)	11 (Aug ‘20 to June ‘21)	0.046	2.1%
	Year 2	11 (July ‘21 to May ‘22)	0.068	
E	Year 1 (initial)	12 (July ‘20 to June ‘21)	0.066	6.3%
	Year 2	11 (July ‘21 to May ‘22)	0.129	
F	Year 1 (initial)	12 (July ‘20 to June ‘21)	0.091	−0.1%
	Year 2	11 (July ‘21 to May ‘22)	0.090	

### 3.4 Are Dingo Activity and Population Density Estimates Correlated?

**FIGURE 6:**
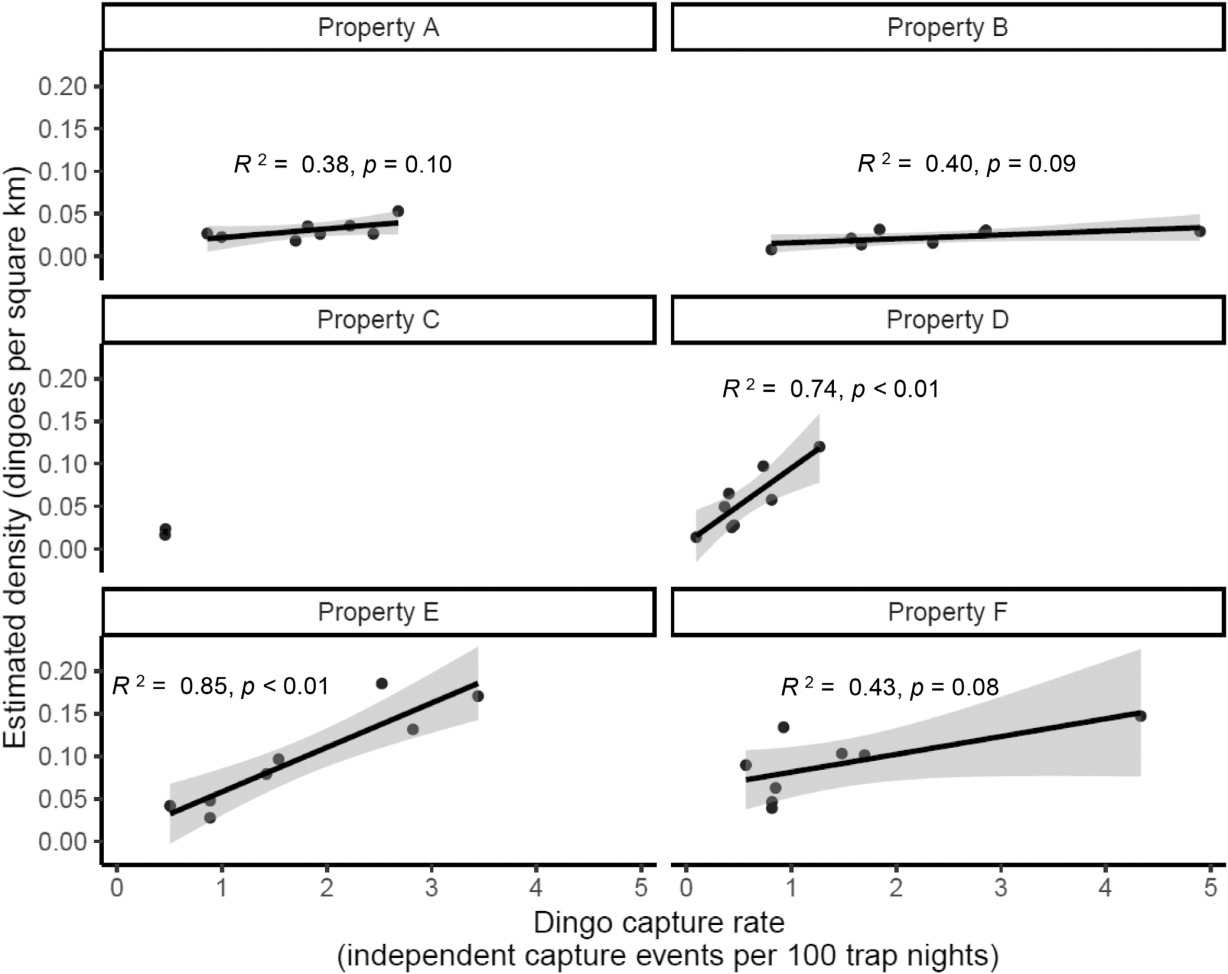
The relationship between capture rate and estimated density of dingoes during the 24‐month survey in six study sites (properties A, B, C, D, E, and F) in the Murchison region of the Southern Rangelands, Western Australia (WA).

Comparisons between dingo activity (number of independent capture events per 100 trap nights) and population density (dingoes per km^2^) at three‐month intervals for each study site are illustrated in Figure 6. There was a strong correlation (*p* < 0.01) between dingo capture rate and density for Properties D and E. The correlations between dingo capture rate and density for Properties A, B, C, and F were weak (*p* > 0.05).

## 4 Discussion

Robust density estimates and knowledge about dingo activity at landscape‐scale cell‐fencing are critical to understanding the effectiveness of dingo population control in the Australian rangelands. The experimental design, spatial scale, and the outcome from this study provide one of the first robust estimates of dingo density at landscape‐scale cell‐fencing. The activities of three prey taxa (macropod, rabbit, and livestock) were significantly correlated with dingo activity, and when these differences were accounted for as covariates, the predicted dingo capture rate significantly decreased with an increase in the number of dingo exclusion fences (from outside the MRVC to inside the MHC). However, these differences in dingo activity were not reflected in differences in actual dingo density, as there was no evidence of an effect of exclusion fencing on the estimated dingo population density. Furthermore, the correlation between dingo activity and density was strong for only two study sites, suggesting differences in behavior across sites.

### 4.1 Is Dingo Activity Correlated With Resource Availability and Exclusion Fencing?

Dingo activity was strongly influenced by prey occurrence. Livestock activity was positively correlated with dingo activity, with the highest overall dingo activity recorded in the northeast sites, which are commercial cattle grazing properties. Similarly, the activity of macropods, which have been reported as the primary constituents of dingo diets in most contexts (Whitehouse 1977; Doherty 2015; Fleming et al. 2022), and rabbits, which are easy‐to‐get prey following rainfall‐driven booms in vegetation (Letnic et al. 2012; Paltridge 2002), were also positively correlated with dingo activity. Rabbits and macropods are the most likely food sources of dingoes in the southwest sites (west and east portions of the Rangelands Park) which have been de‐stocked for over 20 years and are now primarily used for the conservation of biodiversity. The southwest sites have the highest annual rainfall of all six study sites (Bureau of Meteorology 2023), which likely reflects the greater productivity in this part of the study area and the highest rabbit activity compared to other study sites (Figure S1).

Availability of water had no effect on dingo activity. Standing natural water bodies (e.g., lakes, crevices) and artificial water sources (e.g., boreholes, water tanks for commercial grazing) influence species assemblage, especially in the arid/semi‐arid rangelands (Thomson 1992c; Fensham and Fairfax 2008). Therefore, even if dingoes were not reliant on water sources for drinking, dingoes might visit water points for hunting. As such, dingoes have been targeted around livestock waterpoints with the use of poisoned baits (Eldridge et al. 2000; Allen 2012b). However, in this present study, the presence of a permanent water source within 5 km radii of camera trap locations had no effect on dingo activity. Data from this study support the idea that dingoes may not necessarily require free water (Allen 2012a) or the large resource pool from the assemblage of prey species at water points (Thomson 1992c; Brawata and Neeman 2011) to persist in the arid/semi‐arid environment.

### 4.2 Do Dingo Activity and Derived Density Estimates Vary Between Study Sites and Over Time?

Dingo density was highly variable between study sites. The estimated dingo density from this study ranged between 0.019 dingoes per km^2^ for one of the central sites (property C) to 0.100 dingoes per km^2^ for one of the southwest sites (property E). These densities are in the lower range of those estimated across different biomes in Australia (0.010–0.700 dingoes per km^2^) (Gabriele‐Rivet et al. 2019). Compared to the dingo density estimates (0.040–0.147 dingoes per km^2^) from tropical climate zones that comprise a mixture of forests, wetlands, and savanna (Allen et al. 2015; Corbett 1995a; Gabriele‐Rivet et al. 2020), the lower dingo density estimates from this study likely reflect overall lower productivity in the Southern Rangelands of WA, which is characterised by an arid/semi‐arid environment.

Dingo density varied temporally across study sites. Peaks in estimated dingo density across study sites occurred during the period expected to be dingo mating seasons (February to April). All juvenile marked dingoes (i.e., pups) were first detected in camera trap images between October and November. Data from this study is consistent with the literature on dingo breeding season and the emergence of pups from natal dens in arid/semi‐arid environments (Thomson 1992b, 1992c). The general increase in dingo density at the latter end of this study likely reflects the increased productivity of the landscape in the latter stages of the study. The average percentage total vegetation cover [percentage proportion of photosynthetic vegetation (PV) and non‐photosynthetic vegetation (NPV)] assessed monthly within 0.25 km^2^ of each camera trap location (Omogbeme et al. unpublished data) increased during the second year of dingo monitoring in four study sites (Figure S2). This second year of dingo monitoring spanned a *La Niña* period of increasing rainfall in the early months of the year 2022 (Bureau of Meteorology 2023) and therefore presumably increasing productivity.

### 4.3 Is Dingo Management in the Southern Rangelands of WA Effective for Maintenance of Small Livestock?

Despite the marked differences in dingo activity recorded between dingo exclusion fence levels (i.e., from outside the MRVC to inside the MHC), the combined approach of predator‐proof fencing coupled with broadscale bi‐annual coordinated spread of poisoned baits, trapping, and opportunistic shooting at each study site did not have a measurable effect on estimated dingo density across fence levels. The annual mean dingo density estimate across study sites was below 2 dingoes per 100 km^2^ (i.e., 0.02 dingoes per km^2^) at only one study site in the first year, but it was higher across all sites during the second year of monitoring. Therefore, similar to Kennedy et al. (2021), we conclude that the general annual increase in the estimated dingo density in five study sites reflects that the management efforts across the study sites were insufficient to cause a significant reduction in dingo population.

Across Australian rangelands, lethal control of dingo populations within fenced areas is largely by broadscale (ground or aerial) distribution of fresh or manufactured meat containing 1080 [i.e., meat baits (fresh or dried) are infused with 6.0 mg of sodium fluoroacetate in 0.2 mL of standard solution] (Claridge and Mills 2007; Kreplins et al. 2018; Fleming and Parker 1991; Eldridge et al. 2000; Kennedy et al. 2021; Castle et al. 2021). However, dingo baiting programs can be hampered by a combination of factors. First, effective deployments may be hindered by natural or legal confines to where and how poisoned baits can be placed (Fleming et al. 1998; Twigg et al. 2000; Kennedy et al. 2014). Second, non‐target animals such as reptiles, birds, or mammals including other canids interfere with and consume poisoned baits (e.g., McIlroy et al. 1986; Kreplins et al. 2018; Claridge et al. 2006) reducing the number of deployed poisoned baits and the likelihood of bait discovery by dingoes. Third, bait resistance (sensu Allsop et al. 2017) can reduce baiting efficacy. For instance, in the study by Kreplins et al. (2018), dingoes accounted for less than 1% of the total bait uptake, and only young naïve pups took baits while adult dingoes snubbed baits despite their observed activities around baits. There are also likely to be differences in the innate survival instincts of individual dingoes that result in aversive behavior towards baits (Kreplins et al. 2018; McIntyre et al. 2020).

Improving the efficacy of dingo control programs in livestock grazing areas may require consideration of a number of factors. First, ground‐deployment of baits may be less effective than aerial deployment, especially for sites that are inaccessible (see Kennedy et al. 2014). Second, bait presentation (bait type, use of attractant lures, bait positioning, and bait density) has strong effects on bait discovery and uptake by both target and non‐target species (Allen et al. 1989; Glen and Dickman 2003; Hunt et al. 2007; Thomson 1986; Jolly and Jolly 1992; Eldridge et al. 2000; Ballard et al. 2020). Third, the toxicity of poisoned baits at the time of uptake by target species is pivotal (Crawford et al. 2025), as some studies have reported a potential reduction in bait toxicity to sub‐lethal dose after deployment due to leaching or biodegradation (e.g., Fleming and Parker 1991; Saunders et al. 2000; McIlroy et al. 1988; Allsop et al. 2017), which can increase the likelihood of animals developing bait aversion. Lastly, the seasons of deployment strongly determine the age class of dingoes likely to encounter poisoned baits and the bait encounter rates (Allen 2012a; Eldridge et al. 2002), considering geographical variations in the reproductive seasons and biology of dingoes (Thomson 1992b; Catling et al. 1992; Jones and Stevens 1988). Being able to store toxic baits could allow livestock producers to target ‘hot spots’ of predator activity or around specific timing, supplementing or even replacing broadscale baiting, therefore avoiding the potential risk of bait‐resistant populations (Crawford et al. 2025), and supplementing targeted trapping or shooting activity.

### 4.4 Are Dingo Activity and Population Density Estimates Correlated?

Data from this study suggest variation in behavioural responses of dingoes to persecution or confinement by exclusion fencing across the study area. Despite a similar number of individuals, the number of resights of the same individuals (number of identified individuals sighted at more than one camera trap location) was higher in the two study sites outside the MRVC compared to their corresponding adjacent study sites inside the MRVC. Overall, the central sites (properties C and D) had low dingo capture rates and very few resights of the same individuals. This pattern of higher capture rates and resights of individuals in the study sites outside the MRVC (properties A and E) led to marked differences in the calculated effect of exclusion fencing on dingo activity and population density estimates.

The differences in dingo activity and resights can be attributed to differences in the use of vehicular dirt tracks across study sites by the resident dingoes from outside the MRVC to inside the MHC, potentially as a response to persecution. Aside from trapping techniques, other dingo controls (broadscale bi‐annual coordinated spread of toxic baits, targeted baiting, and opportunistic shooting across the landscape) are employed, with these control measures using the same vehicular dirt tracks that were monitored in the present study. The effectiveness of these control measures, e.g., selective removal of bolder naive animals through baits (Allsop et al. 2017) or shooting, is likely to influence dingo behavioural patterns along vehicular dirt tracks.

Variations in dingo activity may not reflect actual population size. The impact of control tools on dingo population has been largely evaluated using dingo activities (such as camera trap rate or indices of track counts i.e., footprints and/or faecal counts) within controlled areas (Fleming 1996a; Twigg et al. 2000; Burrows et al. 2003; Kennedy et al. 2012; Campbell et al. 2019; L. R. Allen 2015; Castle et al. 2021). As such, some studies have reported the recolonisation of controlled areas by dingoes after a prolonged period of reduced or no observed dingo activities (Thomson 1986; Thomson 1992a; Fleming et al. 1996; Eldridge et al. 2000). In the present study, the correlations between dingo capture rate (a measure of dingo activity) and estimated density (a measure of population size from individual identifications) at three‐month intervals for each study suggest that variation in activity may not reflect the actual size of the dingo population. Therefore, reduced or no observed dingo activity during and/or after dingo population control programs may not ascertain a sink in actual dingo population size.

### 4.5 Limitations of This Study

There are caveats to the dingo density estimates in this study, which are associated with the distances between camera traps and between sampling transects. As a logistical and financial reality, a total of 72 camera traps (12 per study site) were used to address the aims of this study. As such, camera traps were mounted 2 km from each other in each transect and transects were spaced at 5–20 km to cover a significant portion of each study site. Although the same 2 km distance between camera traps was used in a single long transect by Gabriele‐Rivet et al. (2020), the shorter transects and more replicates per site implemented in our survey may have influenced the low number of identified individuals sighted at more than one camera trap location and the overall low number of dingoes resighted per camera trap location. While cognisant of these caveats, the estimated dingo capture rates and densities reflect potential differences in behavioural patterns of the dingo populations across the study sites.

## 5 Conclusions

The decoupling between dingo capture rate and dingo density across fence levels (from outside the MRVC to inside the MHC) and in each study site infers that dingoes can be present but less active, presumably as a response to persecution. Considering the huge investment in landscape‐scale exclusion fences and lethal techniques to mitigate the impact of dingoes in the Southern Rangelands of WA, these efforts may have influenced the capture rate of dingoes along vehicular dirt tracks from outside the MRVC to inside the MRVC but did not yield a corresponding effect in dingo density from outside the MRVC to inside the MRVC.

## Author Contributions

**Moses I. Omogbeme:** conceptualization (lead), data curation (lead), formal analysis (lead), investigation (lead), methodology (equal), visualization (lead), writing – original draft (lead). **Malcolm S. Kennedy:** funding acquisition (lead), methodology (equal), supervision (supporting), writing – review and editing (equal). **Tracey L. Kreplins:** formal analysis (supporting), funding acquisition (supporting), methodology (equal), supervision (supporting), writing – review and editing (equal). **Halina T. Kobryn:** formal analysis (supporting), methodology (equal), supervision (supporting), writing – review and editing (equal). **Patricia A. Fleming:** conceptualization (supporting), data curation (supporting), formal analysis (supporting), funding acquisition (supporting), methodology (equal), supervision (lead), writing – review and editing (equal).

## Conflicts of Interest

The authors declare no conflicts of interest.

## Supporting information


Appendix S1.
Appendix S2.

## Data Availability

Data available via the Mendeley Data repository https://data.mendeley.com/datasets/8vs5cyct72 (Omogbeme et al. 2025).
