# Supplementary material for: Activity May Not Reflect the Numbers: An Assessment of Capture Rate and Population Density of Dingoes ( Canis familiaris ) Within Landscape‐Scale Cell‐Fencing
Source: Ecol Evol. 2025 Apr 27;15(4):e71328. doi: 10.1002/ece3.71328 (PMC12034454; doi:10.1002/ece3.71328)
Supplement: Supplementary file 1 — Appendix S1. Appendix S2. [file ECE3-15-e71328-s001.docx]

**APPENDIX**

**Appendix S1. Supporting Methods**

**Experimental design**

Sampling location selection took into consideration the differences in environmental factors such as topography, vegetation type, or standing surface water between the six study sites. To minimise the impact of these differences in the choice of sampling locations, geology, vegetation, as well as pastoral station property boundaries, and the MRVC extensions were mapped using Arc GIS software (version 10.6) to identify parts of the landscape that were suitable for comparison (**Table S1**). The attribute table for the land systems layer was used to develop broad groupings of land systems by geology and vegetation (broadly classified by dominant understory and dominant overstory) for all for all six study sites.

*Rugosity.* Terrain rugosity analysis was undertaken with the Benthic Modeler Tool in ArcMap. Two outputs were initially created: Topographic Position Index (TPI) layer (at the 500 m scale) and the rugosity layer (5 pixels neighbourhood). Both raster files were reclassified and combined, resulting in four values of terrain rugosity: 0 (flat area), 1 (medium), 2 (high), and 3 (very high). The combined raster (hereafter ‘rugosity layer’) was also filtered to exclude minor landscape features (9x9 pixel majority filter) and masked to exclude lakes and buffered (200 m) sand ridges. The rugosity layer was used in the subsequent analyses to exclude lakes and sand ridges.

*Vegetation.* Sampling locations were selected to represent the best grazing areas and therefore were filtered to alluvial plains (excluding salt lakes and breakaways, which were deemed too variable and not expected to be a focus for grazing). Vegetation was also filtered to understorey components consisting of wanderrie grasses, halophytes, or other vegetation (excluding spinifex assuming that they are also not foci for grazing). A database query (‘Geology’ = ‘Plains’) AND (‘Understorey’ = ‘wanderrie grasses’ OR ‘Understorey’ = ‘halophytes’ OR ‘Understorey’ = ‘Other’) was performed to obtain a new information layer called ‘grasses on plains’. To facilitate spatial queries based on tenure and land features, the polygons for both pastoral station boundaries and ‘grasses on plains’ were intersected.

The planned sampling locations, therefore, captured geology as plains (excluding breakaways and salt lakes), and understorey vegetation types (wanderrie grasses, halophytes, or others). The mapped thematic layers of the study area were used to aid on-site analysis of the landscape to identify suitable access and the low-use vehicular dirt roads along which the sampling surveys were carried out in each study site.

| **Table S1:** Datasets, sources, and information derived from these data for pre-survey analysis. | | |
| --- | --- | --- |
| **Type** | **Source** | **Information used for this study** |
| **Raster** |  |  |
| Digital Elevation Model (DEM) | Geoscience Australia (1 second; 30 m pixels), Shuttle Radar Topography Mission (SRTM) | - Slope |
|  |  | - Rugosity |
|  |  | - Topographic Position Index (TPI) |
|  |  |  |
| **Vector** |  |  |
| Roads | Geoscience Australia 1:250k topographic data | - Highway, vehicular dirt tracks |
| Hydrology |  | - Surface water (lakes, springs, rock holes, soak) |
|  |  | - Water course lines |
|  |  | - Man-made water points (native wells, bores/ wind pumps, water tanks) |
| Cadastre | DPIRD | - Pastoral station property boundaries |
| MRVC extensions |  | - Location of dingo-proof fencing |
| Breakaways |  | - Ridges and rocky rises |
| Land systems |  | - Geology (Breakaways, Plains, Salt lakes) |
|  |  | - Understorey vegetation type |
|  |  | - Overstorey vegetation type |

DPIRD: Department of Primary Industries and Regional Development

**Appendix S2. Supporting Discussion**

**Dingo activity (capture rate) was influenced by prey activity (capture rate)**


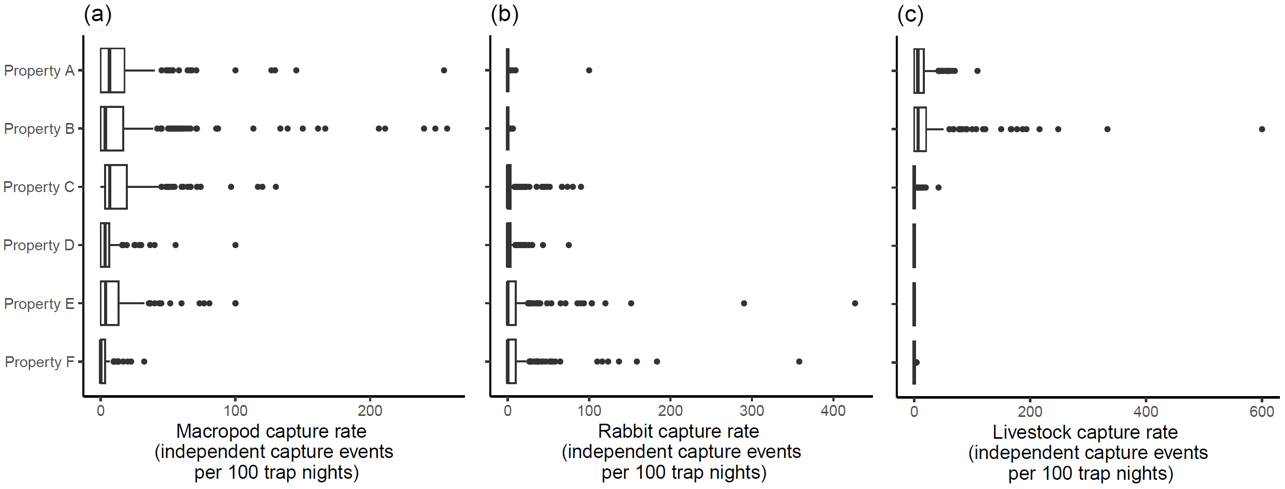


**Figure S1**. Prey group capture rate (independent capture events per 100 trap nights) for dingoes: (a) macropod, (b) rabbit, and (c) livestock in six study sites nested within three dingo exclusion fence level in the Murchison Region of Southern Rangelands, Western Australia (WA) during 24 months of camera trap monitoring.

**Do dingo activity (capture rate) and derived density estimates vary between study sites and over time**


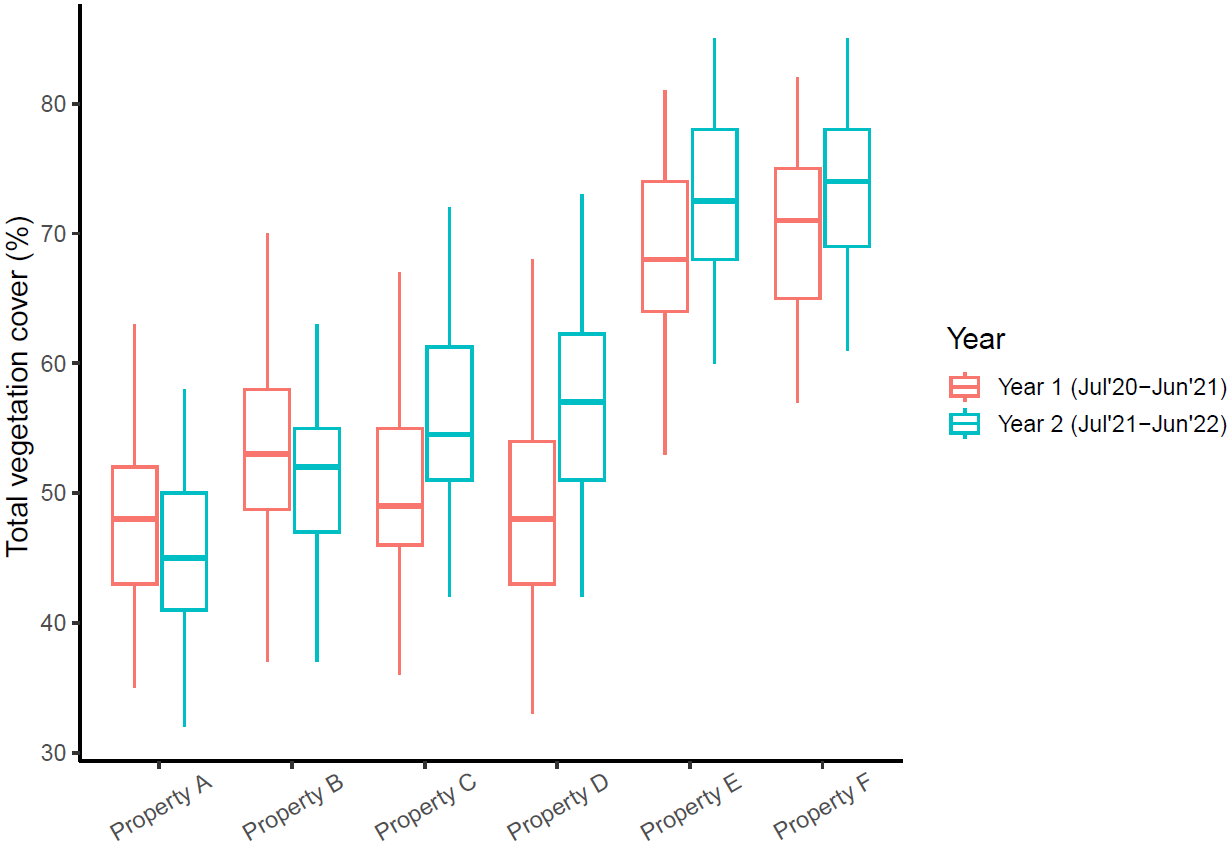


**Figure S2**. Percentage total vegetation cover assessed monthly within 0.25km^2^ of each camera trap location (Omogbeme et al. unpublished data) indicates an increased productivity in four study sites during the second year of dingo monitoring which spanned a *La Niña* period of increasing rainfall in the early months of the year 2022.
